# Lack of the E3 Ubiquitin Ligase March1 Affects CD8 T Cell Fate and Exacerbates Insulin Resistance in Obese Mice

**DOI:** 10.3389/fimmu.2020.01953

**Published:** 2020-08-17

**Authors:** Abdelilah Majdoubi, Jun Seong Lee, Osama A. Kishta, Mohammad Balood, Mohamed Abdelwafi Moulefera, Satoshi Ishido, Sébastien Talbot, Cheolho Cheong, Thierry Alquier, Jacques Thibodeau

**Affiliations:** ^1^Département de Microbiologie, Infectiologie et Immunologie, Université de Montréal, Montreal, QC, Canada; ^2^Département de Pharmacologie et Physiologie, Université de Montréal, Montreal, QC, Canada; ^3^Department of Microbiology, Hyogo College of Medicine, Nishinomiya, Japan; ^4^Institut de Recherches Cliniques de Montréal (IRCM), Montreal, QC, Canada; ^5^Centre de Recherche du Centre Hospitalier de l’Université de Montréal (CRCHUM), Montreal Diabetes Research Center, and Département de Médicine, Université de Montréal, Montreal, QC, Canada

**Keywords:** March1, obesity, T cell metabolism, memory CD8 T cell

## Abstract

Obesity is a major risk factor for the development of insulin resistance and type 2 diabetes. However, the mechanisms that trigger the underlying adipose tissues inflammation are not completely understood. Here, we show that the E3 ubiquitin ligase March1 controls the phenotypic and functional properties of CD8^+^ T cells in mice white adipose tissue. In a diet-induced obesity model, mice lacking March1 [March1 knockout (KO)] show increased insulin resistance compared to their WT counterparts. Also, in obese March1 KO mice, the proportions of effector/memory (Tem) and resident/memory (Trm) CD8^+^ T cells were higher in the visceral adipose tissue, but not in the spleen. The effect of March1 on insulin resistance and on the phenotype of adipose tissue CD8^+^ T cells was independent of major histocompatibility complex class II ubiquitination. Interestingly, we adoptively transferred either WT or March1 KO splenic CD8^+^ T cells into obese WT chimeras that had been reconstituted with Rag1-deficient bone marrow. We observed an enrichment of Tem and Trm cells and exacerbated insulin resistance in mice that received March1 KO CD8 T cells. Mechanistically, we found that March1 deficiency alters the metabolic activity of CD8^+^ T cells. Our results provide additional evidence of the involvement of CD8^+^ T cells in adipose tissue inflammation and suggest that March1 controls the metabolic reprogramming of these cells.

## Introduction

Obesity is associated with a high risk of mortality and morbidity in the world (1). Type 2 diabetes (T2D) and cardiovascular diseases are the most common complications of obesity. Obese adipose tissue (AT) shows signs of inflammation in mice and humans, suggesting that the immune system plays a key role in obesity-related pathologies (2). Many immune cells having an anti-inflammatory role in lean AT switch to a proinflammatory phenotype with obesity (2). The phenotypic change of M2 macrophages to M1 that accompanies the development of obesity is well characterized (3, 4). However, the accumulation of M1 macrophages is preceded by a change in the AT content of T cells (5). Two independent studies have used transgenic mice models of loss-of-function or adoptive transfers to show that CD8^+^ T cells are directly controlling the development of T2D by promoting the accumulation of inflammatory M1-polarized macrophages (6, 7). Interestingly, these white AT (WAT) CD8^+^ T cells produce tumor necrosis factor α (TNF-α) and interferon γ (IFN-γ), which exacerbate insulin resistance (IR) (6).

One of the mechanisms of modulation of the immune response comes from the spatiotemporal diversification of the fate of CD8^+^ T cells (8). Reactivation of tissue resident memory (Trm) CD8^+^ T cells ensures a rapid immune response, and the priming of naive cells sustains this response. However, the conversion of effector cells into a memory quiescent state stops the inflammatory process (9). The diversity of CD8^+^ T cells can vary, depending on the tissue (10, 11). The lymphoid organs contain a large number of naive CD8^+^ T cells, as well as effector and memory CD8^+^ T cells, while WAT is mainly populated with cells with effector and memory phenotypes. In accordance with these observations, different groups have shown that memory T cells in WAT can intervene and protect against infections (12, 13). However, the reactivation of this reservoir of memory cells alters AT physiology (13). One of the characteristics that differ between naive, effector, and memory phenotypes of CD8^+^ T cells is the metabolic pathway used by these subsets of T lymphocytes (10, 14). Naive CD8^+^ T cells are metabolically quiescent and mainly use oxidative phosphorylation to meet their basic energy needs (14, 15). In contrast, effector cells engage both glycolysis and oxidative phosphorylation to meet higher energy needs. At the end of the immune response, the generation of long-lived memory CD8^+^ T cells requires, in the absence of glycolysis, a metabolic reprogramming characterized by the use of extracellular glucose and aims at the synthesis of fatty acids and their breakdown by oxidative phosphorylation (14, 15). Consequently, the pharmacological inhibition of glycolysis in activated CD8^+^ T cells leads to an alteration in the effector functions and promotes the generation of memory T cells (10, 15–18).

Recently, it has been shown that the metabolism of glucose and amino acids plays an essential role in the immune responses induced by T cells. Indeed, Tsai et al. (19) have shown that insulin signaling is important for T-cell proliferation and functions. This effect is mediated by the mTOR-induced activation of the nuclear factor Myc, which genetically controls the expression of the glucose transporter Glut1 and the amino acid transporter CD98. These transporters promote the internalization and degradation of glucose and amino acids during glycolysis and oxidative respiration, respectively (19). Likewise, Ikeda et al. (20) have shown that CD98-mediated uptake of branched-chain amino acids, such as leucine, is important in keeping regulatory CD4^+^ T cell (Treg) in a proliferative state. Again, this effect was found to be linked to the activation of mTOR, which controls the metabolic profile of these cells. Interestingly, both studies have shown that the expression of CD98 and insulin receptor in these cells influence clinical outcome in the context of an influenza infection and autoimmune diseases (19, 20).

The ubiquitination system plays a very important role in regulating the characteristics of immune cells. As a member of the March family of E3-ubiquitin ligases, March1 is highly expressed in antigen-presenting cells (APCs) and downregulates MHC class II molecules (MHCIIs) and CD86 (21). However, basal levels of March1 are detectable in other cells, suggesting that the role of March1 is not limited to ubiquitination of antigen presentation molecules and costimulators in APCs (22). For example, March1 has been shown to target the insulin receptor, TfR and the CD98 heavy chain, which are important for T-cell metabolism (23–25). This led us to hypothesize that March1 could regulate T-cell fate in the context of obesity-induced IR. Here, we show that March1 protects against obesity-induced IR in a CD8^+^ T cells–intrinsic manner. The adoptive transfer of March1-deficient CD8^+^ T cells exacerbated IR in obese Rag knockout (KO) mice, and this is correlated with a higher proportion of cells with an effector phenotype. Finally, absence of March1 in CD8^+^ T cells was associated with high glycolytic activity and increased spare respiratory capacity, which could explain their altered phenotype.

## Materials and Methods

### Mice

Wild-type (WT) C57BL/6J (CD45.2), WT B6.SJL-Ptprca Pepcb/BoyJ (CD45.1) and Rag1 KO (B6.129S7-*Rag1^TM1Mom^*/J) were purchased from Jackson Laboratory. CD45.2^+^ *March1*^–/–^ (March1 KO), and MHCII KI mice were generated by SI (Hyogo College of Medicine, Japan) and backcrossed on the C57BL/6J background (26). They were generously provided by Dr. Paul Roche (National Institutes of Health, Frederick, MD, United States). To avoid any bias from the microbiota or the susceptibility to high-fat diet (HFD)–induced obesity, March1 KO and MHCII KI were further backcrossed to WT C57BL/6J from Jackson Laboratory for five generations, and a colony of WT mice was then bred at the University of Montreal’s animal facility. All the mice were used at the age of 6 to 8 weeks. The housing, breeding, and use of mice were in accordance with guidelines of the University of Montreal’s Animal Care and Use Committee.

### Generation of Bone Marrow Chimeras

WT mice were irradiated twice (4-h interval) with 5 Gy and reconstituted by a tail vein injection of 5 × 10^6^ to 10 × 10^6^ WT (CD45.1), March1 KO (CD45.2), or Rag1 KO bone marrow (BM) cells, as indicated in the text. For mixed BM chimeras (BMCs), a mixture (1:1) of WT (CD45.1), and March1 KO (CD45.2) was injected. BMCs were assigned to control diet (CD) or HFD 6 to 8 weeks after immune cell reconstitution.

### Diet and Insulin Resistance Studies

Mice were fed a control (0.64 kcal/g of fat) or high-fat diet (3.24 kcal/g of fat) (Bio-Serv) according to the indicated periods. All mice were weighed at regular intervals. Accu-check Aviva glucometer and strips were used to measure blood glucose. For insulin tolerance tests (ITTs), fasted (5 h) mice received 0.75 to 1 U/kg of human insulin (Humulin R from Lilly), and blood glucose was measured at the indicated time points.

### T-Cell Transfer Experiments

Spleen CD4^+^ and CD8^+^ T cells were purified using EasySep cell isolation kits (StemCell Technologies). Obese Rag1 KO BMCs received two weekly intravenous injections of 5 × 10^6^ CD4^+^ T cells from WT and 5 × 10^6^ CD8^+^ T cells from either WT or March1 KO mice.

### Single-Cell Preparation for Flow Cytometry

Spleens were pressed in cell strainers using syringe plungers and washed in RPMI 10% fetal bovine serum (FBS). Cells were then incubated in red blood cells (RBCs) lysis buffer for 2 min at room temperature and washed twice prior to staining. Epididymal AT was harvested and minced into small pieces. Single cells were isolated by enzymatic digestion for 20 min at 37°C with gentle shaking using a collagenase mixture containing collagenase I, collagenase XI, and hyaluronidase (Sigma) in Hanks balanced salt solution containing Ca^+^/Mg^+^ and 5% FBS (Wisent), as described (27). After digestion, the single cells and remaining tissue were passed through a 70-μm cell strainer. Cells were then incubated in RBCs lysis buffer for 2 min at room temperature and washed twice prior to staining.

### Metabolic Flux Assay

Isolated naive CD8^+^ splenic T cells from WT or March1 KO mice were used freshly *ex vivo* or activated with plate-bound anti-CD3/CD28 antibodies (1 mg/mL) for 72 h. Cells were seeded at 1.5 × 10^5^ to 1 × 10^6^ cells/well and ECAR and OCR measured using the Seahorse Analyzer (Agilent Technologies).

### Flow Cytometry Analysis

For surface markers, cells were incubated for 10 min in the dark at RT with Zombie (Biolegend) to stain dead cells, washed, and incubated with rat serum (Sigma) to block Fc receptors. Then, cells were washed and incubated on ice for 30 min with mouse-specific antibodies of interest. For the intracellular staining of cytokines, cells were incubated for 2 h with a T-cell activation cocktail (1 μg/mL; Sigma) before labeling surface markers. Then, cells were incubated with fix-perm buffer on ice for 30 min, washed in perm-wash buffer (all from eBioscience), and incubated with the antibodies of interest on ice for 30 min. Acquisitions were performed using the FACS DIVA software on a FACS Canto II or Celesta (BD), and data were processed using FlowJo or Kaluza software.

### Cytometric Bead Array

Stromal vascular fractions (SVFs) were isolated from the whole right epididymal AT pad of obese WT and March1 KO mice as previously described (see *Single-Cell Preparation for Flow Cytometry*). SVFs were lysed in Triton X-100 lysis buffer containing protease inhibitors for 30 min on ice. Cytokines were measured using the Cytometric Bead Array (CBA) mouse inflammation kit (from BD Bioscience). Data were acquired using FACS Canto II instrument and analyzed using FCAP array software (from Soft Flow Hungary Ltd.).

### Statistical Analysis

A two-way analysis of variance (ANOVA) test was used to compare data from different strains under different conditions, whereas a one-way ANOVA followed by Tukey test for multiple comparisons was used to compare results from different strains of mice under the same experimental conditions, *P < 0.05, **P < 0.01, ***P < 0.001.

## Results

### Absence of March1 in Immune Cells Exacerbates Obesity-Induced Insulin Resistance

**FIGURE 1:**
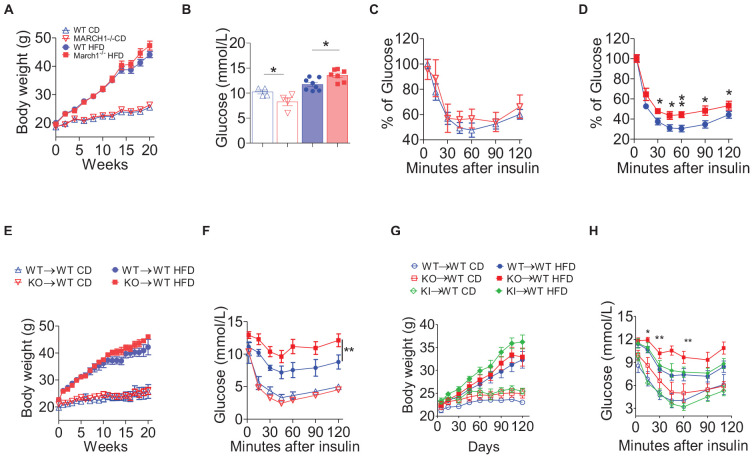
March1 deficiency exacerbates obesity-induced insulin resistance. Mice were fed control diet (CD) or high-fat diet (HFD) and tested for fasting glucose and insulin tolerance (ITT). **(A)** Body weight gain, **(B)** fasting glucose, and **(C,D)** ITT of WT and March1^–/–^ mice after 20 weeks. **(E–G)** Six week old WT mice were lethally irradiated and received an i.v. injection of bone marrow cells from March1^–/–^ or WT mice. After 8 weeks of reconstitution, these WT BMC (WT→WT) and March1-/-BMC (KO→WT) were fed CD or HFD for 20 weeks. **(E)** Body weight gain and **(F)** ITT performed on body weight-matched mice. Experiment was repeated twice. In another independent experiment, MHCII KI BMC (KI→WT) were included. Body weight gain was measured during 16 weeks of feeding **(G)** and body weight-matched mice were submitted to an ITT **(H)**. *P < 0.05 and **P < 0.01.

To study the role of March1 in IR, we first used WT and age-matched March1 KO male mice (28) fed either CD or HFD for 20 weeks. The body weight gain of March1 KO mice was comparable to WT controls in both the CD and HFD groups (Figure 1A). Interestingly, obese March1 KO mice had a significantly higher fasting glucose (FG) than their obese WT counterparts, whereas lean CD-fed March1 KO mice showed a trend toward a decreased FG compared to WT controls (Figure 1B). An ITT performed on these mice revealed that obesity-induced IR was more pronounced in March1 KO compared to WT control mice, whereas no difference was observed between CD-fed mice (Figures 1C,D). Knowing that the lack of March1 increases the expression of insulin receptor (23), the higher IR in obese March1 KO mice could not be explained by the lack of ubiquitination of this receptor in various tissues. However, IR has been associated in many studies with increased inflammation (4, 29, 30).

To verify if the exacerbated obesity-induced IR in March1 KO mice is related to the role of March1 in immune cells, we generated BMCs in which BM cells from WT or March1 KO mice were transferred to lethally irradiated WT recipients to generate WT→WT and KO→WT mice, respectively. After 8 weeks of reconstitution, these BMCs were assigned to CD or HFD for 20 weeks. WT→WT and KO→WT mice showed similar body weight in both CD and HFD groups, apart from a small interval between weeks 12 and 15 where KO→WT mice fed HFD gained transiently more weight. However, this slight difference eventually disappeared at later time points (Figure 1E). To avoid any possible bias coming from obesity, we assessed IR in BMCs that had similar body weight along the entire period of feeding. We found that obese KO→WT mice show a trend toward higher fasting hyperinsulinemia (data not shown) and become more insulin resistant than their body weight–matched WT→WT controls, as shown by ITT (Figure 1F) and glucose tolerance test (data not shown). These results indicate that the effect of March1 on obesity-induced IR is intrinsic to the hematopoietic cell compartment.

March1 is mostly expressed in APCs, including dendritic cells (DCs) and B cells (28, 31). Splenic DCs lacking March1 failed to efficiently present antigens to CD4^+^ T cells and produced less cytokines in response to LPS stimulation (31). These characteristics are related to March1 ubiquitination of MHCIIs but not CD86, another major target of March1 in APCs (32). Knowing that MHCIIs play an important role in AT inflammation (33, 34), we sought to verify whether the exacerbated obesity-induced IR observed in March1 KO mice is related to the lack of ubiquitination of these molecules by March1. To do so, we looked at IR in a March1-sufficient mouse model bearing a mutated I-Aβ chain that cannot be ubiquitinated (MHCII KI) (22). This mouse model should recapitulate the phenotype of March1 KO mice if the effect of March1 is related to the ubiquitination of MHCIIs (33, 34). To avoid any bias from the expression of MHCIIs in non-immune cells, such as adipocytes (34), we generated BMCs by transferring BM cells from WT, March1 KO, or MHCII KI mice to lethally irradiated WT recipients (WT→WT, KO→WT, and KI→WT mice). After 8 weeks of reconstitution, these BMCs were assigned to either CD or HFD for 16 weeks. The three groups of mice developed similar adiposity, as measured by body and the epididymal AT weight (Figure 1G and Supplementary Figure S1). Again, an ITT performed on body weight–matched mice showed that obese KO→WT BMCs developed higher IR compared to obese WT→WT mice, whereas no significant difference was detected in lean BMCs (Figures 1G,H). Interestingly, obese KI→WT mice presented an IR profile that was similar to WT→WT BMCs, indicating that the role of March1 in obesity-induced IR is not related to MHCII-ubiquitination (Figure 1H). Thus, the exacerbation of IR in March1 KO mice is not the result of impaired antigen presentation. Altogether, our results suggest that absence of March1 exacerbates obesity-induced IR by an immune cell–driven mechanism that is independent from the overt accumulation of MHCIIs at the plasma membrane.

### March1 Deficiency Does Not Affect the Phenotypic Switch of AT Macrophages

**FIGURE 2:**
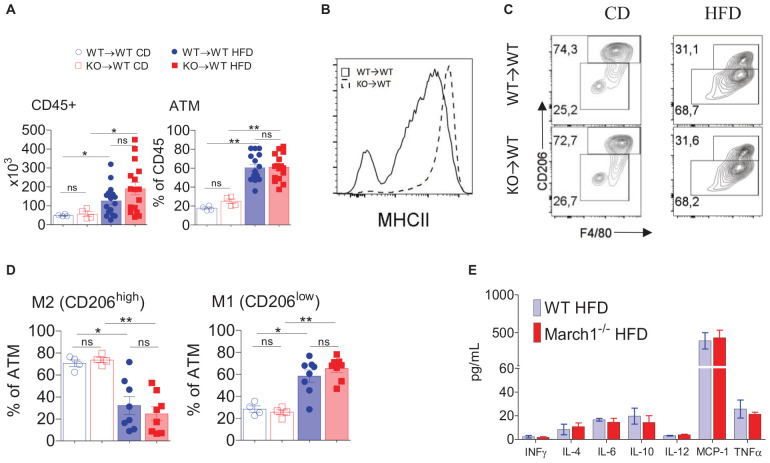
Normal ATM polarization in AT from mice lacking March1. Stromal Vascular Fraction (SVF) cells were isolated by enzymatic digestion of AT from obese and lean BMC. Flow cytometry analysis of SVF for total CD45 + cells **(A-left)** and total ATM proportions **(A-right)**. MHCII surface expression **(B)** was analyzed in ATM from KO→WT and WT→WT obese mice. Representative dot-plots **(C)** and proportions **(D)** of M1 and M2 ATM defined based on the expression of CD206. Cytokine content of total SVF measured by CBA **(E)**. *P < 0.05 and **P < 0.01.

The above-described results demonstrated that the absence of March1 in immune cells exacerbates obesity-induced IR. This effect could be the consequence of AT inflammation mediated by proinflammatory mediators, such as cytokines (2). The role of AT macrophages (ATMs) in obesity and IR is well established. ATM numbers increase with obesity, and they adopt an M1 proinflammatory phenotype instead of the anti-inflammatory M2 phenotype (4). This biased polarization of ATM populations creates an inflammatory milieu in which the secretion of proinflammatory cytokines is enhanced. Some of these cytokines, such as TNF-α and IL-6, directly inhibit the insulin signaling pathway, leading to IR (35–39). Given the expression of March1 in macrophages (40), it is plausible that it could affect ATM polarization. Flow cytometry analysis of AT showed that the total number of immune cells and the percentage of ATMs were similar between lean WT and March1 KO BMCs (Figure 2A, left panel). As expected, obesity increased the number of CD45^+^ cells as well as the percentage of ATMs in WT→WT and KO→WT mice. However, there was no significant difference in terms of AT CD45^+^ cell number and the proportions of ATM between March1 KO and control WT BMCs (Figure 2A, right panel). Interestingly, MHCIIs were up-regulated in ATMs from obese and lean March1 KO compared to WT BMCs, showing that March1 is expressed in these macrophages and that it may affect their polarization (Figure 2B). To verify this, we applied a CD206-based gating strategy that was used by Winer et al. (7) to define M1 and M2 populations in AT. As expected, ATMs in lean mice were predominantly of the M2 phenotype (CD206^hi^), whereas obesity induced a switch toward M1 (CD206^low^) (Figure 2C). Our results indicate that the proportions of M1 and M2 ATMs in KO→WT are comparable to WT→WT mice, suggesting that absence of March1 does not affect macrophage polarization (Figure 2D). To confirm these results, we used a CBA to measure cytokines in the SVF obtained after enzymatic digestion of obese WAT. Consistent with the high proportions of macrophages in AT, the amount of MCP-1 was high in this tissue. We also detected a significant amount of inflammatory cytokines implicated in IR, such as TNF-α and IL-6, as well as the anti-inflammatory cytokine IL-10 (Figure 2E). However, no difference was observed in the level of these cytokines between March1 KO and WT BMCs (Figure 2E). The similar proportions and phenotype of total ATMs, together with the similar cytokine levels in WAT, suggest that the exacerbation of IR in the absence of March1 is not related to its role in the recruitment and phenotypic switch of ATMs.

### March1 Affects the Proportions and Activation Profile of AT CD8^+^ T Cells

**FIGURE 3:**
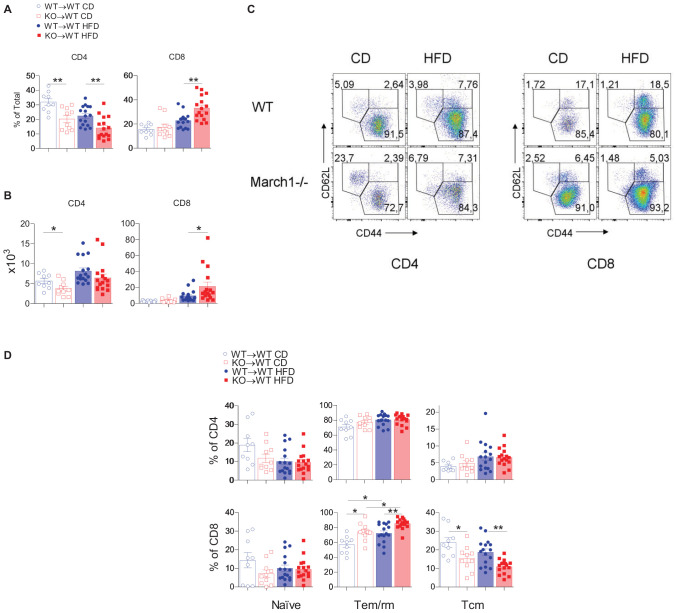
Absence of March1 increases CD8 T cell infiltration and modifies the fate of WAT memory cells. WAT from obese and lean WT→WT and KO→WT mice was analyzed by flow cytometry for T cells. Percentage **(A)** and absolute number **(B)** of CD4^+^ and CD8^+^ T cells. Representative dot plots of naive, Tem/rm and Tcm cells **(C)**. Proportions of naive, Tem/rm and Tcm cells **(D)**. *P < 0.05 and **P < 0.01.

We have shown that ATM polarization is not affected in AT of insulin-resistant March1 KO mice. Importantly, one hallmark of obesity-induced AT inflammation is the increased CD8^+^ to CD4^+^ T-cell ratio (41). CD8^+^ T cells infiltrate AT early after the start of HFD, even prior to ATM recruitment (6). Depletion of CD8^+^ T cells in WT mice protects against obesity-induced IR, and the transfer of CD4^+^, but not CD8^+^ T cells, to Rag1^–/–^ mice improves IR, suggesting that the CD4^+^/CD8^+^ T-cell balance is crucial for AT homeostasis (7). Given the importance of these lymphocytes in AT inflammation and IR, we sought to characterize CD4^+^ and CD8^+^ T cells in AT of March1 KO mice using BMCs. Splenocytes from lean KO→WT and WT→WT mice presented similar proportions and absolute numbers of CD4^+^ and CD8^+^ T cells. In obesity, the proportions of these cells tend to decrease in the absence of March1 with a significant reduction observed in CD4^+^ T cells (Supplementary Figures S3A,B). Interestingly, in AT, a lower percentage of CD4^+^ T cells was observed for both lean and obese KO→WT mice, and the same trend was observed in terms of absolute numbers (Figures 3A,B). By contrast, higher percentages of CD8^+^ T cells were found in AT of obese KO→WT mice, whereas no difference was seen in lean mice. Interestingly, the high percentage of CD8^+^ T cells was reflected in the absolute number of these cells, suggesting that CD8^+^ T cells are enriched specifically in AT of obese KO→WT mice (Figure 3B).

The increased proportions of AT CD8^+^ T cells in the absence of March1 incited us to analyze the phenotype of these cells. Han et al. (13) showed that the majority (>60%) of these cells express CD44, a marker for antigen-experienced T cells, whereas a minor fraction were naive T cells (CD62L^+^CD44^–^). Most CD44^+^ cells were CD62L^–^, suggesting that these cells are effector/memory or resident/memory T cells (Tem/rm). Within CD4^+^ and CD8^+^ T cells, they found that half of the CD44^+^CD62L^–^ population was CD69^+^, indicating that these cells are Trm, whereas the remaining cells were CD69^–^, suggesting that they are Tem. WAT CD8^+^ T cells comprise a population of CD44^+^CD62L^+^ cells analogous to the central memory T cells (Tcm) that are present in high proportions in secondary lymphoid organs (13). To study the phenotype of AT T cells in March1 KO mice, we first verified whether the proportions of CD4^+^ T-cell subsets are affected in the spleen of KO→WT mice using CD44 and CD62L as markers (Supplementary Figure S3C). As expected from the impaired MHCII-mediated antigen presentation in March1-deficient APC, we found decreased proportions of Tem/rm cells and increased proportions of naive cells in the spleen of lean and obese KO→WT mice (Supplementary Figure S3D). This could be due to the disturbed plasma membrane lipid rafts in the presence of high surface MHCIIs (42). In AT, the proportions of all three CD4^+^ T-cell populations were similar between WT→WT mice and KO→WT mice in both obese and lean state (Figures 3C,D). Interestingly, AT from KO→WT mice contained more Tem/rm CD8^+^ cells and less Tcm CD8^+^ cells as compared to WT→WT control BMCs (Figure 3D). Importantly, the proportions of splenic Tem/rm CD8^+^ cells were not affected in the absence of March1 (Supplementary Figure S3D), indicating that the loss of March1 induces a specific enrichment of AT CD8^+^ T cells with an effector/resident memory phenotype, consistent with exacerbated obesity-induced IR in March1 KO mice and KO→WT BMCs.

### Exacerbated IR in March1 KO Mice Is Not Related to the Impaired Generation of Natural Tregs

**FIGURE 4:**
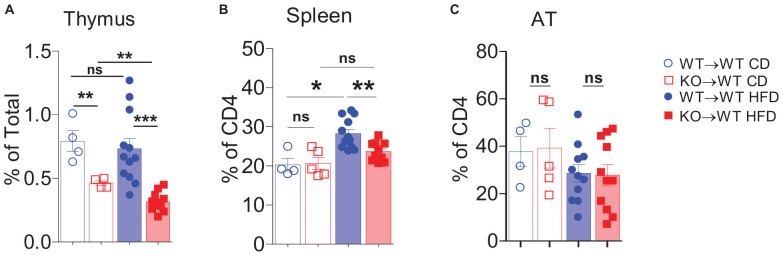
Absence of March1 affects regulatory T cells in thymus and spleen but not in WAT. Thymocytes **(A)**, splenocytes **(B),** and SVF cells from WAT **(C)** of obese and lean WT→WT and KO→WT BMC were analyzed by flow cytometry. Tregs were identified as CD4^+^Foxp3^+^ cells. *P < 0.05 and **P < 0.01.

Tregs play an important role in the attenuation of AT inflammation (43). These cells represent approximately half of the CD4^+^ T-cell compartment in AT of lean mice, and it has been reported that obesity decreases their number, leading to AT inflammation and IR (34). As March1 deficiency results in a 50% reduction of natural Tregs in the thymus (32), we analyzed these cells in AT and spleen of obese and lean WT→WT and KO→WT BMCs. As shown in Figure 4A, consistent with previously reported data (32), we found that Treg proportions in the thymus of lean KO→WT mice were reduced by about half and that this difference was further deepened by obesity. However, the impaired generation of natural Tregs was not reflected by decreased proportions of these cells in the spleens of lean mice (Figure 4B). Obesity induced an increase in splenic Tregs proportions that was significant in WT→WT but not in KO→WT mice. Consistent with previously reported data (33), lean AT from WT BMCs harbored a high amount of Tregs (Figure 4C). Surprisingly, the proportions of Tregs in AT of both lean and obese KO→WT mice were similar to WT→WT controls, suggesting that AT Tregs are not affected by the impaired production of Tregs in the thymus. These results suggest that generation of natural and induced Tregs is differentially affected by March1. Moreover, the normal proportions of Tregs in AT of WT→WT BMCs indicate that the exacerbated obesity-induced IR in these mice is unlikely the result of an impaired generation of natural Tregs.

### Absence of March1 Intrinsically Affects the Activation Profile of CD8^+^ T Cells

**FIGURE 5:**
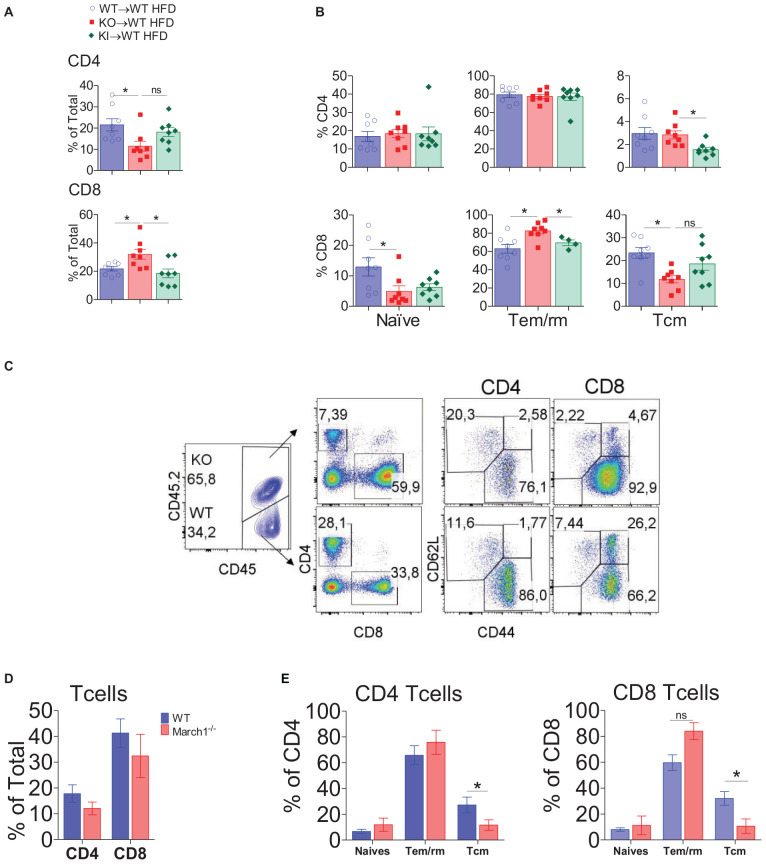
March1 intrinsically affects CD8 T cell infiltration and phenotype in WAT. SVF cells from obese and lean WT→WT, KO→WT and KI→WT BMCs were analyzed by flow cytometry for CD4^+^ and CD8^+^ T cells. Proportions of total **(A)**, naive, effector and memory CD4^+^ and CD8^+^ T cells **(B)**. **(C–E)** Six week old WT mice (*n* = 4) were lethally irradiated and received an i.v injection of a mixture of bone marrow cells from WT (CD45.1) and March1^–/–^ or MHCII KI (CD45.2) mice. After 8 weeks of reconstitution, these mixed BMCs were fed CD or HFD for 20 weeks. Representative dot plots **(C)** and the proportions of total **(D)**, naive, effector and memory CD4^+^ and CD8^+^ T cells from WAT **(E)**. *P < 0.05.

The lack of March1 could alter the phenotype of AT CD8^+^ T cells either indirectly or directly. On the one hand, March1 could play a cell-intrinsic role in CD8^+^ T cells by targeting various molecules, such as CD98 and the insulin receptor, which are implicated in T-cell fate (19, 20). On the other hand, compromised antigen presentation by APCs lacking March1 could indirectly affect the phenotype of CD8^+^ T cells by altering CD4^+^ T-cell helper functions. MHCII KI mice represent a critical tool to verify if the absence of March1 influences CD8^+^ T-cell fate indirectly (44). Indeed, despite the fact that IR in MHCII KI mice was similar to their WT counterpart, we cannot exclude the possibility that the phenotype of their AT CD8^+^ T cells is similar to March1 KO mice. Thus, we analyzed AT from MHCII KI, March1 KO and WT BMCs. Consistent with the above-described data, AT from obese KO→WT mice showed decreased CD4^+^ T cells and increased CD8^+^ T cells percentages, with the proportions of Tem/rm cells being higher only in the CD8^+^ compartment (Figures 5A,B). Interestingly, the percentage of total CD8^+^ T cells and Tem/rm cells in AT from obese KI→WT mice were similar to control WT BMCs (Figures 5A,B). This suggests that the altered CD8^+^ T-cell activation profile observed in AT from March1 KO BMC is not related to the role of March1 in the ubiquitination of MHCIIs. This is in line with a direct role of March1 in CD8^+^ T cells. To further confirm this, we analyzed the phenotype of AT T cells in mixed BMCs that received both CD45.2^+^ March1 KO and CD45.1^+^ WT BM cells in a 1:1 ratio. In these mice, the presence of WT immune cells can compensate any impaired function of March1-deficient APCs. We found a slight but non-significant decrease in the proportions of both CD4^+^ and CD8^+^ T cells within the March1 KO CD45.2^+^ cells, suggesting that the altered proportions of CD4^+^ and CD8^+^ T cells observed in March1 KO mice are somewhat modulated by the presence of WT cells in mixed BMCs (Figures 5C,D). Interestingly, despite this improvement, CD8^+^ T cells lacking March1 still presented higher proportions of Tem/rm cells and lower Tcm as compared to WT CD8^+^ T cells from the same mice (Figures 5C,E). These data suggest that the presence of WT APCs in mixed BMCs cannot rescue the singular phenotype of March1 KO CD8^+^ T cells, indicating a cell-intrinsic role of March1 in the fate of these WAT T cells.

### Transfer of March1-Deficient CD8 T Cells Exacerbates IR

**FIGURE 6:**
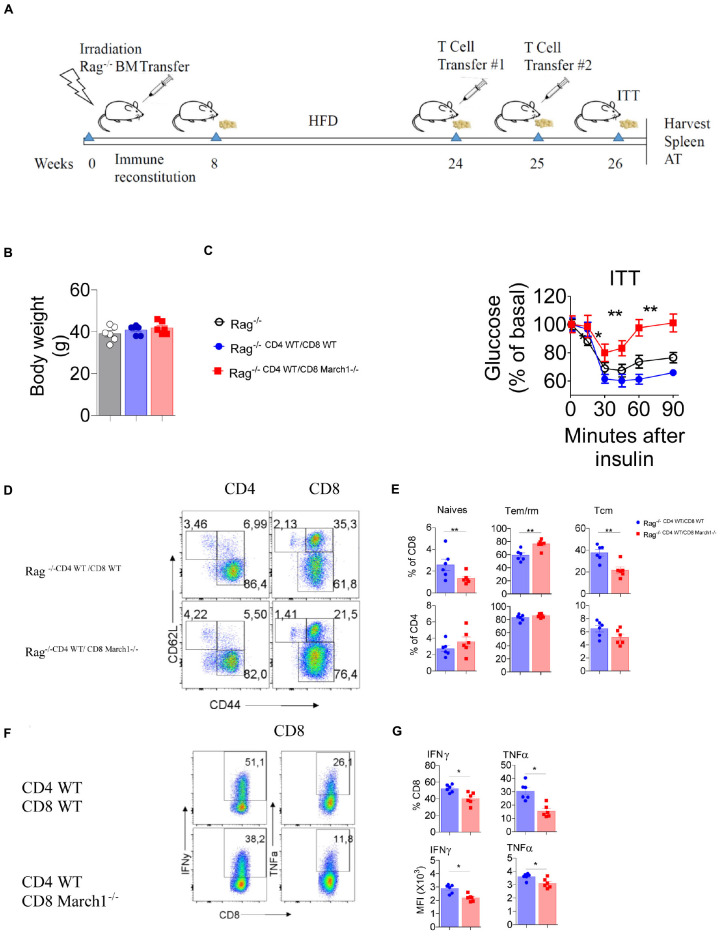
Transfer of CD8^+^ T cells lacking March1 to Rag KO mice exacerbates IR. In order to generate immune cell Rag-deficient mice, 6 week old WT mice were lethally irradiated and received an i.v injection of bone marrow cells from full body Rag deficient mice. After 8 weeks of reconstitution, Rag KO BMC were fed CD or HFD for 16 weeks. At the end of this feeding period, mice having similar body weight **(C)** were injected with sorted 5 × 10^6^ CD8^+^ T cells from 6 to 8 week old March1*-/-* or WT control in combination with 5 × 10^6^ CD4^+^ T cells from WT mice according to the chronology showed in panel **(A)**. T cell-injected and non-injected Rag KO mice **(B)** were submitted to an ITT **(C)**. SVF cells from T cells-injected Rag KO mice were analyzed by flow cytometry for T cell proportions and phenotype. Representative dot plots **(D)** and the proportions **(E)** of naïve, Tem/rm and Tcm CD4^+^ and CD8^+^ T cells. Representative dot plots **(F)**, the proportions of INF-y and TNF-a producing CD8^+^ T cells (G-top) with the MFI (G-bottom) of these cytokines. *P < 0.05 and **P < 0.01.

The fact that March1 deficiency exacerbates IR and that it correlates with an intrinsic change in the CD8^+^ T-cell phenotype raises the question whether the adoptive transfer of CD8^+^ T cells could impact IR (7). Winer et al. (7) have shown that reconstitution of obese Rag-deficient mice with CD4^+^ T cells moderates IR, while CD8^+^ T cells exacerbates it. First, Rag1-deficient BMCs were generated by transferring BM cells from Rag1-deficient mice to lethally irradiated WT recipient mice. After 8 weeks of reconstitution, Rag1-deficient BMCs were fed HFD for 16 weeks, and body weight–matched mice were selected for the adoptive transfer experiment. As Rag1 KO BMC mice on HFD are already highly resistant to insulin, we protected them from IR by transferring WT CD4^+^ T cells (7) together with the CD8^+^ T cells (Figure 6A). Our results indicate that Rag1 KO BMCs that have been reconstituted with WT CD4^+^ T cells together with WT CD8^+^ T cells respond better to insulin than body weight–matched non-reconstituted Rag1 KO BMCs, confirming that CD4^+^ T cells confer protection to Rag KO BMC mice in the presence of CD8^+^ T cells (Figures 6B,C). However, the transfer of March1-deficient CD8^+^ T cells along with WT CD4^+^ T cells worsened IR, suggesting that CD8^+^ T cells lacking March1 are responsible for the exacerbated IR observed above (Figure 6C). To further confirm this result, we analyzed the phenotype of AT CD8^+^ T cells in these mice. Consistent with our previous data, Rag1 KO BMCs that received March1-deficient CD8^+^ T cells have a higher percentage of CD8^+^ Tem/rm cells and lower percentage of CD8^+^ Tcm cells as compared to mice that received WT CD8^+^ T cells (Figures 6D,E). Intriguingly, the ability of March1-deficient CD8^+^ T cells to produce proinflammatory cytokines IFN-γ and TNF-α was decreased (Figures 6F,G). Altogether, our data confirm that the absence of March1 exacerbates IR by increasing the number of WAT CD8^+^ T cells and altering their fate in a cell-intrinsic manner.

### Loss of March1 Increases the Metabolic Activity of CD8^+^ T Cells

**FIGURE 7:**
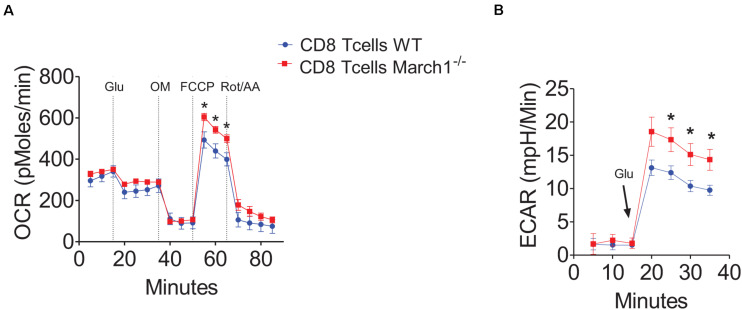
March1 deficiency impairs the metabolic activity of CD8^+^ T cells. Splenic CD8^+^ T cells were purified from March1 KO and WT mice and analyzed using XF24 Seahorse analyzer. **(A)** Spare respiratory capacity measured by oxygen consumption rate (OCR) of naïve CD8^+^ T cells in the presence of glucose (Glu) after Oligomycin (OM), FCCP and Rotenone + Antimycin A (Rot/AA) injection. **(B)** Glycolysis measured by extracellular acidification rate (ECAR) of anti-CD3/anti-CD28 activated CD8^+^T cells after supplementation with glucose (Glu). Experiments were performed using cells from a minimum of two mice and repeated three times with similar results. *P < 0.05.

Our experiments in mixed BMCs and Rag1-deficient mice confirmed that March1 intrinsically affects CD8^+^ T cells to promote IR. This ubiquitin ligase was shown previously to regulate the clonal expansion of CD8^+^ T cells by targeting the amino acid transporter CD98 (45). Moreover, March1 downregulates the insulin receptor, which was shown to control T-cell proliferation and effector functions (19). Interestingly, metabolic reprogramming plays a key role in the determination of CD8^+^ T-cell fate (10, 14). Indeed, T-cell priming requires the switch from oxidative phosphorylation to aerobic glycolysis, and the inhibition of glycolysis facilitates the generation of memory T cells (16, 46, 47). For these reasons, we hypothesized that March1 could regulate the intracellular metabolism of CD8^+^ T cells. Thus, we isolated T cells from March1 KO mice and assessed their glycolytic activity and respiratory capacity in the resting and activated state using the Seahorse extracellular flux analyzer. Our results show that in the absence of March1, the spare respiratory capacity of freshly isolated naive CD8^+^ T cells was increased (Figure 7A). Moreover, the glycolytic activity was increased in anti-CD3 and anti-CD28–activated March1 KO CD8^+^ T cells (Figure 7B). These results indicate that the absence of March1 alters the metabolic reprogramming of both naive and primed CD8^+^ T cells, an effect that might be linked to the altered phenotype observed in AT.

## Discussion

Former studies showed that obesity-induced IR can be triggered by innate immune cells, especially ATMs (3, 4). However, other observations confirmed the implication of adaptive immunity, especially T cells, in IR (6, 7). In fact, CD8^+^ T-cell infiltration even precedes macrophages recruitment in AT and is sufficient to trigger IR (6). The role of CD4^+^ T cells remains controversial. Winer et al. (7) showed that CD4^+^ T cells protect Rag-deficient mice from IR mainly by the immune suppressive effect of Tregs. However, absence of MHCII antigen presentation molecules, which are critical for activation of antigen specific CD4^+^ T cells, protects against obesity-induced IR (33, 34). Interestingly, the specific lack of MHCIIs in macrophages is sufficient to recapitulate this effect, suggesting that innate immunity and adaptive immunity are acting together to trigger IR (33). The expression of March1 in both the innate and the adaptive immune cells and its role in antigen presentation suggested to us that this ubiquitin ligase is involved in the pathogenesis of obesity-induced IR. Indeed, others have recently shown that aging affects glucose tolerance in March1 KO mice (48). We found that IR is exacerbated in the full-body March1 KO mice and in BMCs lacking March1 only in hematopoietic cells. CD8^+^ T-cell infiltration in WAT was more important, and so was the proportion of cells with an effector/resident phenotype within these cells. In mice bearing both March1-sufficient and -deficient immune cells, the percentage of cells with an effector/resident phenotype was also higher in March1-deficient CD8^+^ T cells as compared to WT control CD8^+^ T cells, indicating an intrinsic role of March1. Transfer of splenic CD8^+^ T cells from March1 KO mice exacerbates IR in obese Rag1 KO recipient BMCs, confirming the implication of March1 in the cell-autonomous pathogenic proprieties of CD8^+^ T cells.

Although CD8 T-cell infiltration in AT was shown to trigger IR, the functional characteristics that support the pathological role of these cells remain unclear (6). However, recently, two studies from the groups of Han et al. (13) and Misumi et al. (12) suggested that these cells display phenotypic characteristics of Tem and Trm effector populations. Indeed, they express CD44, the marker of antigen experience, but lack CD62L. Consistent with their effector functions, cytotoxic CD8^+^ T lymphocytes represent a reservoir of pathogen-specific memory T cells that have a critical role in immune responses against infections as well as in inflammation and tissue remodeling of visceral AT (12, 13). Upon rechallenge infection, the recall of these memory cells alters AT homeostasis and reduces survival of obese but not lean mice (12, 13). These reports indicate that the effector functions of these cells trigger AT inflammation and IR in obesity. A dominant fraction of these cells expressed T-bet and had the potential to produce the cytokines IFN-γ and/or TNF-α (13). *Ex vivo* ligation of CD3/CD28 for 72 h revealed the presence of granzyme B–producing CD8 + T cells in lean AT, and interestingly, obesity induced a fourfold increase in the proportion of these cells (29). However, our results showed that freshly isolated CD8 T cells from AT of lean WT and March1 KO mice are able to produce IFN-γ and TNF-α but not granzyme B after PMA stimulation (Figure 6F and data not shown). The reason behind the pathogenesis of these AT cells is still unknown, but one possible explanation could be the presence of some autoreactive CD8 T-cell clones. Indeed, AT-associated T cells were shown to display a biased TCR Vα repertoire, which is suggestive of an antigen-specific expansion (7, 29). Moreover, obesity was shown to reshape the visceral AT-derived MHC class I immunopeptidome and to generate antigenic peptides capable of driving CD8^+^ T-cell responses (49). A peptide derived from lactate dehydrogenase A (LDHA) or B chain, named LDHA_237__–__244_, was identified as an obese VAT-specific immunogenic peptide that was capable of eliciting proinflammatory CD8^+^ T-cell responses (49). This means that obesity makes the environment of AT auspicious for the recruitment and the effector functions of such clones. The metabolic environment of AT can have a significant effect on the functions of lymphocytes at least in part through their metabolic programming (13). For example, insulin signaling was recently shown to decrease the number and the function of AT regulatory T cells (50).

Here, we strengthened the evidence for the implication of CD8^+^ T cells in AT inflammation and showed that a phenotypic switch in a large proportion of these cells correlated with the development of obesity-induced IR in mice. In our study, a disturbed phenotype of T cells is triggered by absence of March1, which is expressed at low level in T cells compared to other cells, such as APCs (32). Interestingly, the best characterized targets of this E3 ubiquitin ligase, for instance, MHCII and CD86, are not expressed in murine T cells (44), suggesting that other March1 targets, such as insulin receptor and CD98, may prevent CD8^+^ T cells from becoming pathogenic. Importantly, these two targets are known to control T-cell metabolism. The amino acid transporter CD98 regulates the proliferation and immune functions of T cells trough the activation of mTOR (20). Similar to CD98, insulin receptor boosts the proliferation and the effector functions of T cells (19). This effect is related to the metabolic role of insulin receptor on the activation of mTOR and the induction of Myc, a nuclear factor responsible for the expression of glucose and amino acid transporters, including CD98 (19). Internalization and engagement of glucose and amino acids in glycolysis and oxidative phosphorylation promote cytokine production and cell proliferation, respectively (46, 47, 51, 52). However, the absence of insulin receptor and CD98 has distinct outcomes in terms of cytokine production. While insulin receptor deficiency in T cells decreased both metabolism and IFN-γ production, lack of CD98 decreased the metabolism but increased the production of IFN-γ (19, 20). Interestingly, surface expression of CD98 is higher in CD8^+^ T cells lacking March1 or bearing a mutated CD98 that cannot be ubiquitinated (45). The increased expression of CD98 in these cells was correlated with increased proliferation, suggesting that ubiquitination of CD98 by March1 affects CD8^+^ T cell biology (45). Accordingly, our results show that the metabolic activity is increased, and the IFN-γ production is decreased in CD8^+^ T cells lacking March1.

Although CD8^+^ T cells induce the M1 polarization of ATMs (6), our results indicate that the lack of March1 in CD8^+^ T cells exacerbates obesity-induced IR without affecting the relative proportions of M1 and M2 macrophages. In the CD206-based gating strategy, ATMs showed two populations, CD206^high^ and CD206^neg–low^. Only CD206^high^ cells secrete IL-10, a phenotype reminiscent of M2 macrophages, whereas the remaining cells produce MCP-1 and TNF-α, indicating that these cells adopt an M1 phenotype (7). Thus, the lack of March1 seems to have no effect on ATM polarization, a finding supported by the similar concentrations of MCP-1, TNF-α and IFN-γ as well as other cytokines measured in WT and March1-deficient mice. On the other hand, intracellular measurement of IFN-γ and TNF-α using flow cytometry showed that the absence of March1 decreased the ability of CD8^+^ T cells to produce these cytokines. These data suggest that IFN-γ and TNF-α are not a prerequisite in the exacerbation of IR by CD8^+^ T cells. Future experiments will aim at clarifying how CD8^+^ T cells exacerbate IR in absence of March1. Mechanistically, we demonstrated that the lack of March1 increased both the glycolytic activity and respiratory capacity of CD8^+^ T cells. This intrinsic role of March1 in the regulation of CD8^+^ T-cell metabolism may result from its ability to ubiquitinate transmembrane molecules, such as CD98 and/or insulin receptor. Future studies will address the impact of these T-cell fate variations when WAT CD8^+^ memory cells are solicited to protect the host against infectious challenges (12, 13).

## Dedication

We dedicate this work to the memory of CC who passed away in the early phase of this work.

## Data Availability Statement

The raw data supporting the conclusions of this article will be made available by the authors, without undue reservation.

## Ethics Statement

The animal study was reviewed and approved by the Comité de déontologie de l’expérimentation sur les animaux (CDEA) de l’Université de Montréal.

## Author Contributions

AM and JT conceived the project. AM, JL, and OK performed the experiments and analyzed the data. MB, MM, and ST contributed to the T cells adoptive transfer experiments. TA provided expertise with assessment of glucose and insulin tolerance. SI generated the March1 KO and MHCII KI mice. AM wrote the manuscript under the supervision of JT.

## Conflict of Interest

The authors declare that the research was conducted in the absence of any commercial or financial relationships that could be construed as a potential conflict of interest.
