# Supplementary material for: Lack of the E3 Ubiquitin Ligase March1 Affects CD8 T Cell Fate and Exacerbates Insulin Resistance in Obese Mice
Source: Front Immunol. 2020 Aug 17;11:1953. doi: 10.3389/fimmu.2020.01953 (PMC7461985; doi:10.3389/fimmu.2020.01953)
Supplement: Supplementary file 1 [file Data_Sheet_1.pdf]

Supplemental figures

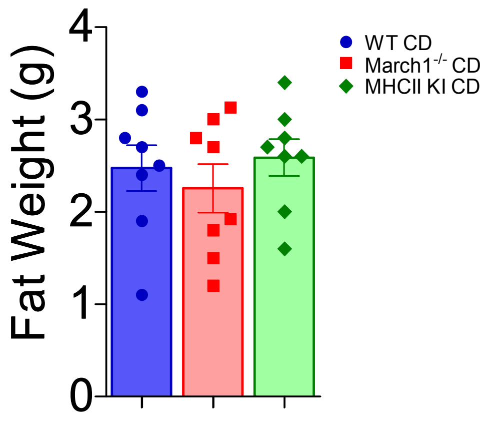

Figure 1S: Epididymal AT weight of BMC. Obese mice in figure 1H was sacrificed and epididymal AT were extracted and weighed.

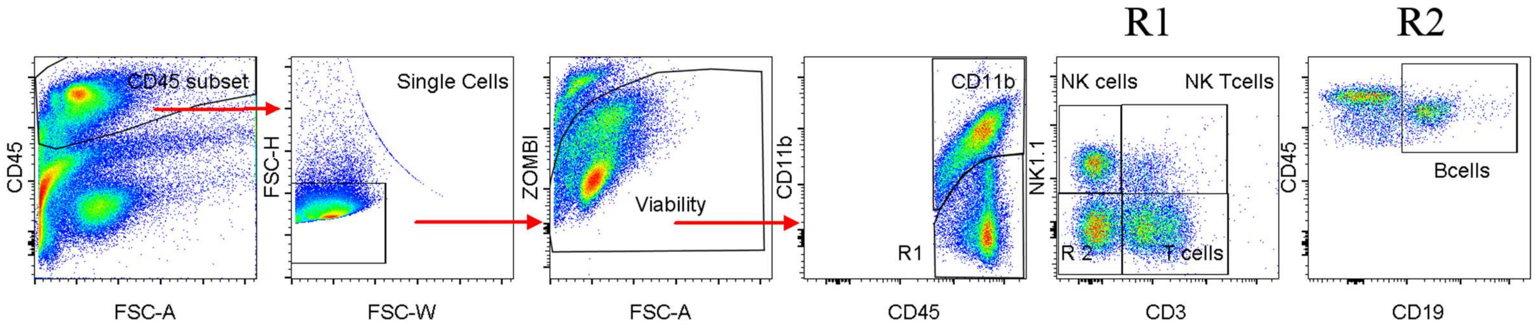

Figure 2S: Gating strategy of immune cells immune in AT

SVF cells from AT of obese and lean WT and March1$^{-/-}$ BMC were analysed by flow cytometry. Single and live immune cells were defined as CD45^{+}. After excluding CD11b^{+} cells, the NK cells, NK T cells, T cells and B lymphocytes were defined using NK1.1, CD3 and CD19 markers.

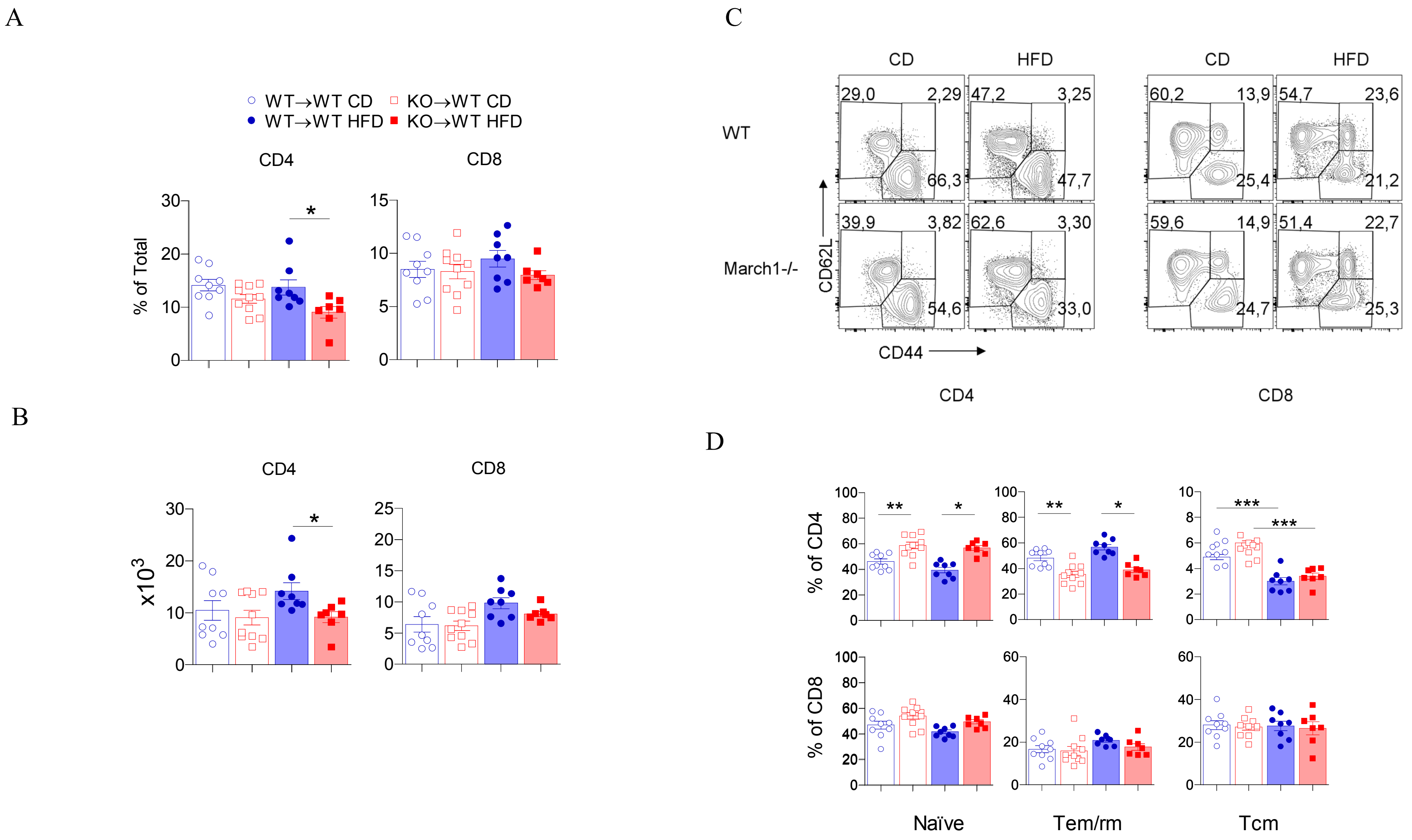

Figure 3S: Absence of March1 alters the activation phenotype of CD4 but not CD8 T cells in the spleen.
Spleen from lean and obese WT and March1$^{-/-}$ BMC was analysed by flow cytometry for T cells. Percentage (A) and absolute number (B) of CD4^{+} and CD8^{+} T cells. Representative dot plots of naive, Tem/rm and Tcm cells (C). Proportions of naive, Tem/rm and Tcm cells (D).
